# Analysis of Detection Enhancement Using Microcantilevers with Long-Slit-Based Sensors

**DOI:** 10.3390/s130100681

**Published:** 2013-01-07

**Authors:** Abdul-Rahim A. Khaled, Kambiz Vafai

**Affiliations:** 1 Mechanical Engineering Department, King Abdulaziz University, Jeddah 21589, Saudi Arabia; 2 Mechanical Engineering Department, University of California, Riverside, CA 92521, USA

**Keywords:** microcantilever, slit, detection, deflection, enhancement, disturbance

## Abstract

The present work analyzes theoretically and verifies the advantage of utilizing rectangular microcantilevers with long-slits in microsensing applications. The deflection profile of these microcantilevers is compared with that of typical rectangular microcantilevers under the action of dynamic disturbances. Various force-loading conditions are considered. The theory of linear elasticity for thin beams is used to obtain the deflection-related quantities. The disturbance in these quantities is obtained based on wave propagation and beam vibration theories. It is found that detections of rectangular microcantilevers with long-slits based on maximum slit opening length can be more than 100 times the deflections of typical rectangular microcantilevers. Moreover, the disturbance (noise effect) in the detection quantities of the microcantilever with long-slits is found to be always smaller than that of typical microcantilevers, regardless of the wavelength, force amplitude, and the frequency of the dynamic disturbance. Eventually, the detection quantities of the microcantilever with long-slits are found to be almost unaffected by dynamic disturbances, as long as the wavelengths of these disturbances are larger than 3.5 times the microcantilever width. Finally, the present work recommends implementation of microcantilevers with long-slits as microsensors in robust applications, including real analyte environments and out of laboratory testing.

## 1. Introduction

The recent advances in nanoscience and nanotechnologies have led to the development of innovative and highly sensitive microsensors. These microsensors are now becoming pivotal tools in exploring many chemical and biological phenomena. An example of these sensors is the microcantilever. The deflection of the microcantilever was first used for atomic force microscopy [1]. Microcantilevers are now being used universally to accurately assay unknown species present in a medium [2–4]. Moreover, they can be used to monitor the presence of specific diseases inside a human body at early stages [5]. The sensing feature of the microcantilevers is generally based on measuring the deflection caused by the adhesion of some specific species, called either analyte or target, on the receptor coating layer of the microcantilever [6–9]. The analyte-receptor adhesion produces compression/tension surface stress, thus bending of the microcantilever occurs, causing the microcantilever to deflect [10]. The adherence-induced deflections can be measured using optical techniques [11,12] or using electric signals in the case of piezoresistive microcantilevers [6,7]. These deflections frequently range from a few tens to a few hundreds of nanometers [10–12]. Accordingly, increasing the sensitivity of microcantilever detection is a major challenge when it is used to detect or monitor low concentrations of analyte.

The detection capability of the microcantilever is influenced by the disturbance level in the adjacent medium. Fritz *et al.* [10] indicated that the deflection of the microcantilever due to external excitations could reach 5–10 times the microcantilever deflection due to analyte-receptor adhesion. The basic constituents of these excitations are the flow disturbances, acoustic wave disturbances and variations in the microcantilever thermal conditions prior to and after injection of the analyte solution. The flow disturbances and acoustic wave disturbances are usually called dynamic disturbances. Further developments in microcantilever technology were carried out so that the deflection signal due to the microsensing effect can be magnified, therefore, the microsensing deflection signal can be easily distinguished from the disturbance (noise) in the deflection signals [13–17]. Consequently, Khaled *et al.* [6] emphasized the necessity to design special microcantilever assemblies for this purpose. Many of these assemblies have been analyzed and validated [14,18]. Moreover, additional innovative methods for enlarging the deflection signal due to microsensing effects were proposed and discussed [9,19–22]. Some of these methods are based on controlling both the geometry of the fluidic cell incubating the microcantilevers and their geometrical distribution.

A remarkable microcantilever assembly among the assemblies proposed in the work of Khaled *et al.* [6] is the rectangular microcantilever with a long-slit. The adherence-induced detection of this type of microcantilevers is almost unaffected by the dynamic disturbances [6]. This type of micocantilever assemblies are made of rectangular microcantilevers with the receptor coating being placed on one half of the upper surface of the microcantilever and along the opposite half of the lower surface of the microcantilever. Furthermore, this microcantilever has a long slit along the interface between the receptor coating and the remaining uncoated surface portion (portion free from receptor). The long-slit allows the separated sides of the microcantilever to have deflections in opposite directions upon analyte adhesion with the receptors. These deflections are able to produce slit opening lengths (normal to the deflection axis) that are much larger than the deflections of typical rectangular microcantilevers. The slit opening length, which can be correlated to analyte concentration, is affected less by dynamic disturbances [6]. This is because both surfaces are subjected to almost similar flow drags or similar acoustic waves. To the best of the authors' knowledge, no additional works have been conducted to demonstrate these aspects. As such, analysis of deflections of rectangular microcantilever with long-slits is the main objective of the present work.

In this work, the advantage of utilizing the rectangular microcantilever with long-slit established by Khaled *et al.* [6] in microsensing applications is explored theoretically. Various force loading conditions that can produce noticeable deflections such as the concentrated force, and prescribed surface stress due to analyte adhesion are considered. The linear elasticity theory for thin beams [23] is used to obtain the deflections and the different detection quantities like the maximum slit opening length and the maximum opening width. The dynamic disturbance is considered to have a harmonic wave form acting on the points of major deflections with force amplitude proportional to square of disturbance frequency [6]. Different detection indicators are defined and various dimensionless controlling parameters are identified. The performance of rectangular microcantilevers with long-slits is compared with the performance of typical rectangular microcantilevers. This is in order to map out conditions that produce magnification of the sensing deflection with minimum disturbance in the deflection.

## 2. Theoretical Analysis

### 2.1. The Typical Rectangular Microcantilever

**Figure 1.**
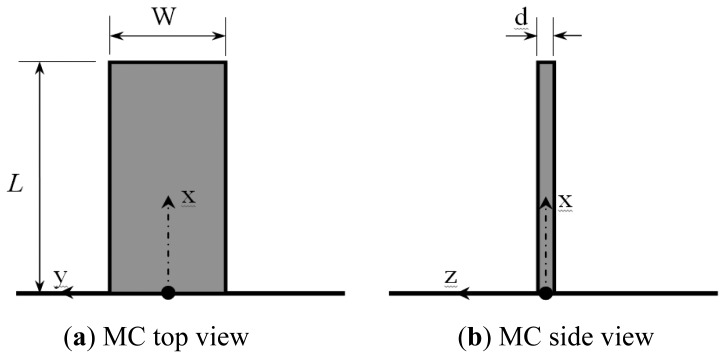
Schematic diagrams and the corresponding coordinate system for typical rectangular microcantliever (MC): (**a**) Top view of rectangular MC; and (**b**) Side view of rectangular MC.

The geometry of the typical rectangular microcantilever considered in this work is shown in Figure 1(a,b). The properties of this microcantilever are given by the extension length *L*, width *W*, thickness *d*, Young's modulus *E* and Poisson's ratio *ν*.

#### 2.1.1. Deflections of the Typical Rectangular Microcantilever

When the length of the microcantilever is much larger than its width, Hooke's law for small deflections can be used to relate the microcantilever deflection at a given cross-section to the effective elastic modulus *Y* of the microcantilever and the bending moment *M* acting on that section [23]. It is given by:

(1) $$\frac{d^{2}z}{dx^{2}}=\frac{M}{YI}$$

where *I* is the area moment of inertia of the microcantilever cross-section about its neutral axis. It is given by:

(2) $$I=\frac{1}{12}Wd^{3}$$

The boundary conditions for Equation (1) are given by:

(3a,b) $$z\left(x=0\right)={\frac{dz}{dx}\;|}_{x=0}=0$$

The magnitude of microcantilever stress at bottom surface (*z* = *d*/2) or upper surface (*z* = −*d*/2), *σ*, associated with the bending moment *M* can be calculated from the following equation:

(4) $$\sigma =\frac{M}{I}\left(\frac{d}{2}\right)$$

##### 2.1.1.1. Concentrated Force Loading

If a concentrated force in the direction of the *z*-axis is exerted on the microcantilever tip located at *x* = *L*, then the internal bending moment *M* at any cross-section is linearly increasing from the tip to the base *x* = 0. The internal bending moment distribution is equal to:

(5) $$M=FL\left(1-\frac{x}{L}\right)$$

For this case, the effective elastic modulus is the same as the elastic modulus (*Y* = *E*). The magnitude of maximum stress occurs at (*x,z*) = (0, ±*d*/2). It is denoted by *σ_oF_*. Using Equation (4), *σ_oF_* can be shown to be equal to:

(6) $$\sigma_{oF}=\frac{6FL}{Wd^{2}}$$

The solution of Equation (1), denoted by *z_F_*(*x*), can be expressed as:

(7) $$z_{F}\;\left(x\right)=\left(\frac{6FL^{3}}{EWd^{3}}\right)\;\left[{\left(\frac{x}{L}\right)}^{2}-\frac{1}{3}{\left(\frac{x}{L}\right)}^{3}\right]$$

The maximum deflection (*z_F_*)*_max_* which occurs at *x* = *L* can be expressed as:

(8) $${\left(z_{F}\right)}_{\max}=\frac{4FL^{3}}{EWd^{3}}$$

We define the concentrated force deflection indicator *Z_F_* as the ratio of the maximum microsensor deflection per maximum stress under constant concentrated force applied at the microcantilever tip. Using Equations (6) and (8), *Z_F_* can be shown to be equal to:

(9) $$Z_{F}\equiv \frac{{\left(z_{F}\right)}_{\max}}{\sigma_{oF}}=\left(\frac{2}{3}\right)\frac{L^{2}}{Ed}$$

##### 2.1.1.2. Prescribed Differential Surface Stress

When one side of the microcantilever is coated with a thin film of receptor, the microcantilever will bend if the analyte molecules adhere on that layer. This adhesion causes a difference in the surface stresses across the microcantilever cross-section (Δ*σ*). This results in an internal bending moment *M* at each cross-section. *M* is related to Δ*σ* through the following equation [2,23]:

(10) $$M=\frac{\Delta \sigma Wd}{2}$$

For this case, the effective elastic modulus varies with the elastic modulus according to the following relationship:

(11) $$Y=\frac{E}{1-\nu }$$

Δ*σ* can be considered to vary along the microcantilever length according to the following relationship:

(12) $$\Delta \sigma =\Delta \sigma_{o}\;{\left(\frac{x}{L}\right)}^{n}$$

where *n* is the model index. This variation is expected as the analyte concentration in the surrounding environment increases as the distance from the microcantilever base increases. As such, the solution of Equation (1) denoted by *z*_Δ_*_σ_*(*x*) subject to boundary conditions given by Equation 3(a,b) can then be expressed as:

(13) $$z_{\Delta \sigma }\left(x\right)=6\left(\frac{1}{n^{2}+3n+2}\right)\times \left(\frac{1-\nu }{E}\right)\Delta \sigma_{o}{\left(\frac{L}{d}\right)}^{2}{\left(\frac{x}{L}\right)}^{n+2}$$

The maximum deflection due to analyte adhesion is obtained from Equation (13) by substituting *x* = *L*. It is equal to:

(14) $$z_{\Delta \sigma \;max}=6\left(\frac{1}{n^{2}+3n+2}\right)\times \left(\frac{1-\nu }{E}\right)\Delta \sigma_{o}{\left(\frac{L}{d}\right)}^{2}$$

Equation (14) is reducible to Stoney's equation when *n* is set to be equal to *n* = 0. We define the prescribed surface stress deflection indicator *Z*_Δ_*_σ_* as the ratio of the maximum microsensor deflection per maximum differential surface stress under the given prescribed surface stresses. Using Equations (12) and (14), *Z*_Δ_*_σ_* can be shown to be equal to:

(15) $$Z_{\Delta \sigma }\equiv \frac{{\left(z_{\Delta \sigma }\right)}_{\max}}{\Delta \sigma_{o}}=6\left(\frac{1}{n^{2}+3n+2}\right)\times \left(\frac{1-\nu }{E}\right){\left(\frac{L}{d}\right)}^{2}$$

#### 2.1.2. The Disturbance in the Deflections of the Typical Rectangular Microcantilever

The one degree of freedom model that can best describe the disturbance in the tip deflection of the typical rectangular microcantilever, *z_d_*, is shown in the following differential equation [6,24,25]:

(16) $$m_{\text{eff},1}\frac{d^{2}z_{d}}{dt^{2}}+k_{\text{eff},1}z_{d}=P_{o}\omega^{2}\times \text{cos}\left(\omega t\right)$$

where *m_eff_*,_1_ is the effective mass of the microcantilever at its tip and *k_eff_*,_1_ is the effective stiffness of the microcantilever at its tip. *ω* is the frequency of the dynamic disturbance force and *t* is time variable. *P_o_* is the effective amplitude of the dynamic disturbance force at the tip per square of the frequency of dynamic disturbance. Equation (16) is based on the assumption that the microcantilever is excited in the first mode of vibration and that excitations occur without total energy dissipation. *m_eff_*,_1_ and *k_eff_*,_1_ are given by the following expressions[24]:

(17) $$m_{\text{eff},1}=\frac{33}{140}\rho \text{WdL}$$

(18) $$k_{\text{eff},1}=\left(\frac{1}{4}\right)EW{\left(\frac{d}{L}\right)}^{3}$$

where *ρ* is the density of the microcantilever. The particular solution of the differential equation given by Equation (16) is the following:

(19) $$z_{d}=\left\{\frac{{\left(\omega \;/\omega_{o}\right)}^{2}}{1-{\left(\omega \;/\omega_{o}\right)}^{2}}\right\}\left(\frac{140}{33}\right)\left(\frac{P_{o}}{\rho \text{WdL}}\right)\times \cos\left(\omega t\right)$$

where *ω_o_* is the first mode natural frequency which is equal to [24]:

(20) $$\omega_{o}=1.0299\left(\frac{d}{L^{2}}\right)\sqrt{\frac{E}{\rho }}$$

The total maximum deflection of the typical rectangular microcantilever, *z_t_*, which is the sum of the deflection due to loading of the microcantilever plus the disturbance in the deflection, can be mathematically expressed as follows:

(21) $$\left[z_{t,F},z_{t,\Delta \sigma }\right]=\left[{\left(z_{F}\right)}_{\max}+z_{do},{\left(z_{\Delta \sigma }\right)}_{\max}+z_{do}\right]$$

where *z_do_* is given by:

(22) $$z_{do}=\left\{\frac{{\left(\omega \;/\omega_{o}\right)}^{2}}{\left|1-{\left(\omega \;/\omega_{o}\right)}^{2}\right|}\right\}\left(\frac{140}{33}\right)\left(\frac{P_{o}}{\rho \text{WdL}}\right)$$

Define the clearness indicator of the microsensor deflection signal (*χ_D_*) as the ratio of maximum deflection due to loading type *D*, where *D* can be *F* or Δ*σ* loading types, to the sum of that deflection plus the amplitude of the maximum disturbance in the deflection. As such, *χ_F_* and *χ*_Δ_*_σ_* can be shown to be equal to:

(23) $$\chi_{F}=\frac{\left|1-{\left(\omega \;/\omega_{o}\right)}^{2}\right|}{\left|1-{\left(\omega \;/\omega_{o}\right)}^{2}\right|+{\left(\omega \;/\omega_{o}\right)}^{2}\left(\frac{P_{o}{\omega_{o}}^{2}}{F}\right)}$$

(24) $$\chi_{\Delta \sigma }=\frac{\left|1-{\left(\omega \;/\omega_{o}\right)}^{2}\right|}{\left|1-{\left(\omega \;/\omega_{o}\right)}^{2}\right|+\left(\frac{2}{3}\right)\left(n^{2}+3n+2\right){\left(\omega \;/\omega_{o}\right)}^{2}\times \left(\frac{P_{o}\omega_{o}^{2}\left(L\;/d\right)}{\Delta \sigma_{o}W\left[1-\nu \right]}\right)}$$

### 2.2. The Rectangular Microcatilever with a Long-slit

**Figure 2.**
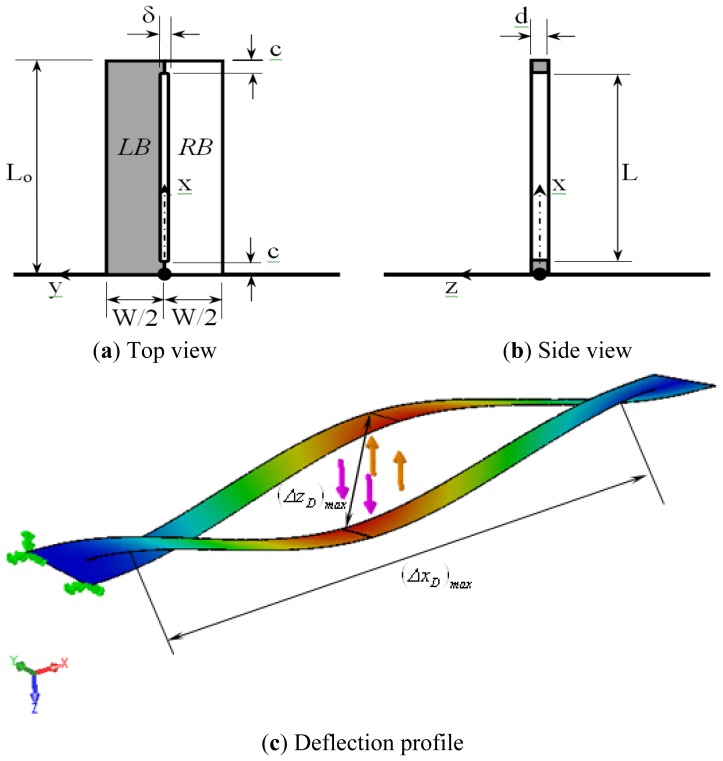
Schematic diagrams and the corresponding coordinate system for the rectangular microcantliever with long-slit: (**a**) Top view of rectangular MC with long-slit; (**b**) Side view of rectangular MC with long-slit, and (**c**) Deflection profile of rectangular MC with long-slit with major deflection quantities.

The geometry of the rectangular microcatilever with long-slit is shown in Figure 2(a,b). The thickness of the microcantilever and the slit is *d*. The slit width is *δ* while its length is *L* where *δ* ≪ *L*. The microcantlever length is *L_o_*. It is larger than the slit length by 2*c* as shown in Figure 1(c,d). The side beams on left and right sides of the slit have same width which is equal to *W*/2. Each side beam has an area moment of inertia *I* given by:

(25) $$I=\frac{1}{24}Wd^{3}$$

#### 2.2.1. Deflections of the Rectangular Microcatilever with Long-slit

The length of the microcantilever with long-slit is considered to be much larger than its width. As such, Hooke's law for small deflections can be used to relate the microcantilever deflection at a given cross-section to the effective elastic modulus *Y* of the microcantilever and the internal bending moment *M* acting on that section [23]. It is given by Equation (1).

##### 2.2.1.1. Concentrated Force Loadings

Let the middle cross-section of the beam on the left side of the long-slit be loaded by a normal concentrated force of magnitude *F* and in the direction of the positive *z*-axis. On the other hand, the beam on the right side of the long-slit is considered to be loaded by a normal concentrated force of magnitude *F*, but in the direction of the negative *z*-axis. Practically, this loading configuration can be attained by coating one surface of the left side beam of the long-slit with a receptor coating while coating the opposite surface of the right side beam of the long-slit with a similar receptor coating layer. Furthermore, the analyte of this configuration is considered to be electrically charged with one type of electrical charges. As such, forces of equal magnitudes with opposite directions are induced after application of appropriate alternating electrical fields on the adhered analyte molecules on the receptor coatings. When *c* ≤ *x* ≤ *c* + *L*/2, the internal bending moment *M* distributions on the left side beam (LB) and the right beam (RB) can be shown to be equal to the following:

(26) $$M=\left(\frac{1}{8}\right)FL\times [\begin{array}{cc} 1+4\left(\frac{c}{L}-\frac{x}{L}\right), & \text{for}\;\text{LB}\\ 4\left(\frac{x}{L}-\frac{c}{L}\right)-1, & for\;RB \end{array},c\leq x\leq c+L\;/2$$

Accordingly, Equation (1) changes to the following:

(27) $$\frac{d^{2}z_{F}}{dx^{2}}=3\left(\frac{FL}{EWd^{3}}\right)\times [\begin{array}{cc} 1+4\left(\frac{c}{L}-\frac{x}{L}\right), & \text{for}\;\text{LB}\\ 4\left(\frac{x}{L}-\frac{c}{L}\right)-1, & for\;RB \end{array},c\leq x\leq c+L\;/2$$

The boundary conditions of Equation (27) are given by:

(28a–b) $$\begin{array}{l} \text{Left}\;\text{beam}:z_{F}\left(x=c\right)={\frac{dz_{F}}{dx}\;|}_{x=c}=0\\ \text{Right}\;\text{beam}:z_{F}\left(x=c\right)={\frac{dz_{F}}{dx}\;|}_{x=c}=0 \end{array}$$

The magnitude of the maximum stress occurs at (*x,z*) = (*c*,±*d*/2). It is denoted by *σ_oF_*. Using Equation (4), *σ_oF_* can be shown to be equal to:

(29) $$\sigma_{oF}=\left(\frac{3}{2}\right)\frac{FL}{Wd^{2}}$$

The solution of Equation (1), denoted by *z_F_*(*x*), can be expressed as:

(30) $$z_{F}=3\left(\frac{FL^{3}}{EWd^{3}}\right)\times [\begin{array}{cc} \left(\frac{2}{3}\right)\left\{{\bar{c}}^{3}-{\bar{x}}^{3}\right\}+\left(\frac{1}{2}\right)\left\{{\bar{c}}^{2}+{\bar{x}}^{2}\right\}-\bar{c}\bar{x}\left\{1+2\bar{c}-2\bar{x}\right\}, & \text{for}\;\text{LB}\\ \left(\frac{2}{3}\right)\left\{{\bar{x}}^{3}-{\bar{c}}^{3}\right\}-\left(\frac{1}{2}\right)\left\{{\bar{c}}^{2}+{\bar{x}}^{2}\right\}+\bar{c}\bar{x}\left\{1+2\bar{c}-2\bar{x}\right\}, & \text{for}\;\text{RB} \end{array}$$

where *x̄* = *x/L* and *c̄* = *c/L*. The maximum deflections of LB and RB which occurs at *x* = *c* + *L*/2 can then be found. They are equal to the following:

(31) $${\left(z_{F}\right)}_{\max}=\left(\frac{1}{8}\right)\left(\frac{FL^{3}}{EWd^{3}}\right)\times [\begin{array}{cc} 1, & \text{for}\;\text{LB}\\ -1, & \text{for}\;\text{RB} \end{array}$$

If the position of RB above the concentrated load is taken as the datum of the rectangular microcantilever with long-slit, then the maximum deflection in that microcantilever denoted by (Δ*z_F_*)*_max_* (see Figure 2c) will be:

(32) $${\left(\Delta z_{F}\right)}_{\max}={{\left(z_{F}\right)}_{\max}\;|}_{LB}-{{\left(z_{F}\right)}_{\max}\;|}_{RB}=\left(\frac{1}{4}\right)\left(\frac{F}{EW}\right){\left(\frac{L}{d}\right)}^{3}$$

The deflection of LB and RB in the positive *z*-direction and negative *z*-direction, respectively, causes an opening in the long-slit along the *x-z* plane. This opening has its maximum width along the z-axis equals to (Δ*z_F_*)*_max_*. The opening maximum length along the *x*-axis denoted by (Δ*x_F_*)*_max_* (see Figure 2c) can be obtained by the following equation:

(33) $${\left(\Delta x_{F}\right)}_{\max}={\left\{L-2\left({x|}_{z_{F}=d/2}-c\right)\right\}|}_{LB}={\left\{L-2\left({x|}_{z_{F}=-d/2}-c\right)\right\}|}_{LB}$$

By using Equation (30) and the solution of the cubic equation, (Δ*x_F_*)*_max_* can be shown to be equal to the following:

(34) $$\begin{array}{cc} \frac{{\left(\Delta x_{F}\right)}_{\text{ma}x}}{L}=\cos\left(\frac{1}{3}\left[2\pi -\cos^{-1}\left(-1+\frac{d}{{{\left(z_{F}\right)}_{\max}\;|}_{LB}}\right)\right]\right)+\frac{1}{2}, & {{\left(z_{F}\right)}_{\max}\;|}_{LB}\geq \frac{d}{2} \end{array}$$

As (Δ*x_F_*)*_max_* > (Δ*z_F_*)*_max_* when (*z_F_*)*_max_*∣*_LB_* ≫ *d*/2, the concentrated force deflection indicator *Z_F_* of the microcantilever with long-slit can be redefined as the ratio of (Δ*x_F_*)*_max_* per maximum stress. It is equal to:

(35) $$Z_{F}\equiv \frac{{\left(\Delta x_{F}\right)}_{\max}}{\sigma_{oF}}=\frac{L}{{{\left(z_{F}\right)}_{\max}\;|}_{LB}}\left(\frac{1}{8}\right)\left(\frac{L^{2}}{Ed}\right)\left\{1+\left(\frac{2}{3}\right)\times \cos\left(\frac{1}{3}\left[2\pi -\cos^{-1}\left(-1+\frac{d}{{{\left(z_{F}\right)}_{\max}\;|}_{LB}}\right)\right]\right)\right\}$$

Define the first detection enhancement indicator of the rectangular microcantilever with log-slit due to concentrated force loading *γ_1,F_* as the ratio of *Z_F_* indicator of the rectangular microcantilever with log-slit to the *Z_F_* indicator of the typical rectangular microcantilever. As such, *γ_1,F_* is equal to:

(36) $$\gamma_{1,F}=\left\{\frac{d}{{{\left(z_{F}\right)}_{\max}\;|}_{LB}}\right\}\left(\frac{L}{d}\right)\left(\frac{3}{16}\right)\left\{1+\left(\frac{2}{3}\right)\times \cos\left(\frac{1}{3}\left[2\pi -{\text{cos}}^{-1}\left(-1+\frac{d}{{{\left(z_{F}\right)}_{\max}\;|}_{LB}}\right)\right]\right)\right\}$$

The maximum values of (*Z_F_*)*_max_*∣*_LB_* / *d* that produces *γ_F_* ≤ 1.0 are shown in Table 1.

##### 2.2.1.2. Prescribed Differential Surface Stress

When one surface of LB is coated with a thin film of receptor, it will bend if analyte molecules adhere on that layer due to induced tension/compression surface stress. This adhesion causes a difference in the surface stresses across the microcantilever section (Δ*σ*). On the other hand, RB will bend in the opposite direction if the receptor coating is placed on the surface opposite to that of the LB coated surface. The relation between the magnitude of the internal bending moment *M* at each cross-section of LB and RB [2,23] and Δ*σ* is given by Equation (14). Let Δ*σ* be considered to vary along the microcantilever length according to the following relationship:

(37) $$\Delta \sigma =\Delta \sigma_{o}{\left(\frac{x}{L}-\frac{c}{L}\right)}^{n}$$

The effective elastic modulus for this case is shown in Equation (15). Accordingly, Equation (1) changes to the following:

(38) $$\frac{d^{2}z_{\Delta \sigma }}{dx^{2}}=6\left(\frac{1-\nu }{Ed^{2}}\right)\Delta \sigma_{o}{\left(\frac{x}{L}-\frac{c}{L}\right)}^{n}\times [\begin{array}{cc} 1, & \text{for}\;\text{LB}\\ -1, & \text{for}\;\text{RB} \end{array}$$

The boundary conditions of Equation (38) are given by:

(39a–b) $$\begin{array}{l} \text{Left}\;\text{beam}:z_{\Delta \sigma }\left(x=c\right)={\frac{dz_{\Delta \sigma }}{dx}\;|}_{x=c}=0\\ \text{Right}\;\text{beam}:z_{\Delta \sigma }\left(x=c\right)={\frac{dz_{\Delta \sigma }}{dx}\;|}_{x=c}=0 \end{array}$$

The solution of Equation (1) can be expressed as:

(40) $$z_{\Delta \sigma }\left(x\right)=6\left(\frac{1}{n^{2}+3n+2}\right)\times \left(\frac{1-\nu }{E}\right)\Delta \sigma_{o}{\left(\frac{L}{d}\right)}^{2}{\left(\bar{x}-\bar{c}\right)}^{n+2}\times [\begin{array}{cc} 1, & \text{for}\;\text{LB}\\ -1, & \text{for}\;\text{RB} \end{array}$$

where *x̄* = *x/L* and *c̄* = *c/L*. If the position of the midsection of RB is taken as the datum of the rectangular microcantilever with long-slit, then the maximum deflection in that microcantilever denoted by (Δ*z*_Δ_*_σ_*)*_max_* will be:

(41) $${\left(\Delta z_{\Delta \sigma }\right)}_{\max}={{\left(z_{\Delta \sigma }\right)}_{\max}\;|}_{LB}-{{\left(z_{\Delta \sigma }\right)}_{\max}\;|}_{RB}=3{\left(\frac{1}{2}\right)}^{n}\left(\frac{1}{n^{2}+3n+2}\right)\times \left(\frac{1-\nu }{E}\right)\Delta \sigma_{o}{\left(\frac{L}{d}\right)}^{2}$$

The deflection of LB and RB in the positive *z*-direction and negative *z*-direction, respectively, causes an opening in the long-slit along the *x-z* plane. This opening has its maximum width along the z-axis equals to (Δ*z_F_*)*_max_*. The opening maximum length along the *x*-axis denoted by (Δ*x_F_*)*_max_* can be obtained by the following equation:

(42) $${\left(\Delta x_{\Delta \sigma }\right)}_{\max}={\left\{L-2\left({x\;|}_{z_{\Delta \sigma }=d/2}-c\right)\right\}\;|}_{LB}={\left\{L-2\left({x\;|}_{z_{\Delta \sigma }=-d/2}-c\right)\right\}\;|}_{LB}$$

By using Equation (40) and the solution of the quadratic equation, (Δ*x*_Δ_*_σ_*)*_max_* can be shown to be equal to the following:

(43) $$\frac{{\left(\Delta x_{\Delta \sigma }\right)}_{\max}}{L}=1-2{\left\{\frac{\left(n^{2}+3n+2\right)}{12}\times \left[\frac{Ed/\Delta \sigma_{o}}{\left(1-\nu \right){\left(L/d\right)}^{2}}\right]\right\}}^{\frac{1}{n+2}}$$

As (Δ*x*_Δ_*_σ_*)*_max_* > (Δ*z*_Δ_*_σ_*)*_max_* when (*Δx_Δσ_*)*_max_*∣*_LB_* ≫ *d*/2, the concentrated force deflection indicator *Z*_Δ_*_σ_* of the microcantilever with long-slit can be redefined as the ratio of (Δ*x*_Δ_*_σ_*)*_max_* per maximum stress. It is equal to:

(44) $$Z_{\Delta \sigma }\equiv \frac{{\left(\Delta x_{\Delta \sigma }\right)}_{\max}}{\Delta \sigma_{o}}=\frac{1-{\left(\frac{d}{2{{\left(z_{\Delta \sigma }\right)}_{\max}\;|}_{LB}}\right)}^{\frac{1}{n+2}}}{\Delta \sigma_{o}/L}$$

Define the first detection enhancement indicator of the rectangular microcantilever with long-slit due to prescribed differential surface stress loading *γ*_Δ_*_σ_* as the ratio of *Z*_Δ_*_σ_* indicator of the rectangular microcantilever with log-slit to the *Z*_Δ_*_σ_* indicator of the typical rectangular microcantilever. As such, *γ*_Δ_*_σ_* is equal to:

(45) $$\gamma_{\Delta \sigma }={\left(\frac{1}{2}\right)}^{n+1}\left(\frac{L}{d}\right)\left(\frac{d}{2{{\left(z_{\Delta \sigma }\right)}_{\max}\;|}_{LB}}\right)\left\{1-{\left(\frac{d}{2{{\left(z_{\Delta \sigma }\right)}_{\text{ma}x}|}_{LB}}\right)}^{\frac{1}{n+2}}\right\}$$

The maximum values of (*z*_Δ_*_σ_*)*_max_*|*_LB_/d* that produces *γ*_Δ_*_σ_* ≤ 1.0 are shown in Table 2.

#### 2.2.2. The Disturbance in the Deflections of the Rectangular Microcantilever with Long-slit

The one degree of freedom model that can best describe the disturbance in the deflections at the midsections of LB and EB of the rectangular microcantilever of the long-slit, *z_d_*, is shown in the following differential equation [6,24,25]:

(46) $$m_{\text{eff},2}\frac{d^{2}z_{d}}{dt^{2}}+k_{\text{eff},2}z_{d}=P_{o}\omega^{2}[\begin{array}{l} \cos\left(\omega t\right),\;\text{for}\;\text{LB}\\ \cos\left[\pi \left(W/\lambda \right)-\omega t\right],\;\text{for}\;\text{RB} \end{array}$$

where *m_eff_*,_2_ is the effective mass of the LB or RB at their midsections, *k_eff_*,_2_ is the effective stiffness of the LB or RB at their midsections and *P_o_* is the effective amplitude of the dynamic disturbance force at LB or RB midsections per square of disturbance frequency and *ω* is the frequency of the dynamic disturbance force. The variable *t* and quantity *λ* are the time variable and the wavelength of the dynamic disturbance, respectively. Equation (46) is based on the assumption that the microcantilever is excited in the first mode of vibration without total energy dissipation. *m_eff_*,_2_ and *k_eff_*,_2_ can be shown to be equal to the following [24]:

(47) $$m_{\text{eff},2}=0.19179\;\rho \text{WdL}$$

(48) $$k_{\text{eff},2}=8EW{\left(\frac{d}{L}\right)}^{3}$$

The particular solution of the differential equation given by Equation (46) is the following:

(49) $$z_{d}=5.2140\left\{\frac{{\left(\omega \;/\omega_{s}\right)}^{2}}{1-{\left(\omega \;/\omega_{s}\right)}^{2}}\right\}\left(\frac{P_{o}}{\rho \text{WdL}}\right)\times [\begin{array}{l} \cos\left(\omega t\right),\;\text{for}\;\text{LB}\\ \cos\left[\pi \left(W/\lambda \right)-\omega t\right],\;\text{for}\;\text{RB} \end{array}$$

where *ω_s_* is the first mode natural frequency which is equal to:

(50) $$\omega_{s}=6.4585\left(\frac{d}{L^{2}}\right)\sqrt{\frac{E}{\rho }}=6.2710\omega_{o}$$

Thus, the total maximum deflection of LB and RB denoted by *z_tL_* and *z_tR_*, respectively, is the sum of the deflection due to force loadings of these beams plus the disturbance in the deflection at time equal to *t* = 0. They can be mathematically expressed as follows:

(51) $$\left[\begin{array}{c} z_{tL,F}\\ z_{tL,\Delta \sigma } \end{array}\right]=\left[\begin{array}{c} {\left(z_{FL}\right)}_{\max}+z_{d1}\\ {\left(z_{\Delta \sigma R}\right)}_{\max}+z_{d1}\cos\left[\pi \left(W/\lambda \right)\right] \end{array}\right]$$

where *z_d1_* is given by:

(52) $$z_{d1}=5.2140\left\{\frac{{\left(\omega \;/\omega_{s}\right)}^{2}}{\left|1-{\left(\omega \;/\omega_{s}\right)}^{2}\right|}\right\}\left(\frac{P_{o}}{\rho \text{WdL}}\right)$$

Since the position of the midsection of RB is taken as the datum of the rectangular microcantilever with long-slit, the maximum total deflection in that microcantilever denoted by (Δ*z_t,F_*)*_max_* or (Δ*z_t,Δσ_*)*_max_* will be:

(53) $$\left[\begin{array}{c} {\left(\Delta z_{t,F}\right)}_{\max}\\ {\left(\Delta z_{t,\Delta \sigma }\right)}_{\max} \end{array}\right]=\left[\begin{array}{c} {{\left(z_{t,F}\right)}_{\max}\;|}_{LB}-{{\left(z_{t,F}\right)}_{\max}\;|}_{RB}\\ {{\left(z_{t,\Delta \sigma }\right)}_{\max}\;|}_{LB}-{{\left(z_{t,\Delta \sigma }\right)}_{\max}\;|}_{RB} \end{array}\right]=\left[\begin{array}{c} {\left(\Delta z_{F}\right)}_{\max}\\ {\left(\Delta z_{\Delta \sigma }\right)}_{\max} \end{array}\right]+z_{d1}\left(1-\cos\left[\pi \left(W/\lambda \right)\right]\right)\times \left[\begin{array}{c} 1\\ 1 \end{array}\right]$$

By inspection of Equations (21) and (53), the maximum ratio of the amplitude of the disturbance in the deflection of the rectangular microcantilever with a long-slit to that of the typical microcantilever is lower than 0.01 when the wavelength satisfies the following constraints:

(54) $$\begin{array}{ccc} \frac{1}{2m+0.28713}<\left(\frac{\lambda }{W}\right)<\frac{1}{2m-0.28713}, & m=1,2,3,\ldots \infty ; & \left(\frac{\lambda }{W}\right)>3.4827 \end{array}$$

The clearness indicator of the deflection signal of the present microsensor (*χ_D_*) is redefined here as the ratio of maximum deflection due to loading type *D*, where *D* can be *F* or Δ*σ* loading types, to the sum of that deflection plus the maximum disturbance in the deflection. As such, *χ_F_* and *χ*_Δ_*_σ_* for this case are equal to:

(55) $$\chi_{F}=\frac{\left|1-0.02543{\left(\omega \;/\omega_{o}\right)}^{2}\right|}{\left|1-0.02543{\left(\omega \;/\omega_{o}\right)}^{2}\right|+0.5{\left(\omega \;/\omega_{o}\right)}^{2}\left(\frac{P_{o}\omega_{o}^{2}}{F}\right)\left(1-\cos\left[\pi \left(W/\lambda \right)\right]\right)}$$

(56) $$\chi_{\Delta \sigma }=\frac{\left|1-0.02543{\left(\omega \;/\omega_{o}\right)}^{2}\right|}{\left|1-0.02543{\left(\omega \;/\omega_{o}\right)}^{2}\right|+\left(\frac{1}{3}\right)\left(2^{n-3}\right)\left(n^{2}+3n+2\right){\left(\omega \;/\omega_{o}\right)}^{2}\left(\frac{P_{o}\omega_{o}^{2}\left(L/d\right)}{\Delta \sigma_{o}W\left[1-\nu \right]}\right)\left(1-\cos\left[\pi \left(W/\lambda \right)\right]\right)}$$

To compare between the clearness indicator of the rectangular maicrocantilever with long-slit and that of typical microcantilever, the second detection enhancement indicator (*γ_2,D_*) is defined as the ratio of *χ_D_*-value of the rectangular microcantilever with long-slit to that of the typical rectangular microcantilever where *D* can be either *F* or Δ*σ*. Mathematically, they are equal to the following:

(57) $$\gamma_{2,F}=\frac{\left|1-0.02543{\left(\omega \;/\omega_{o}\right)}^{2}\right|\left\{\left|1-{\left(\omega \;/\omega_{o}\right)}^{2}\right|+{\left(\omega \;/\omega_{o}\right)}^{2}C_{1}\right\}}{\left|1-{\left(\omega \;/\omega_{o}\right)}^{2}\right|\left\{\left|1-0.02543{\left(\omega \;/\omega_{o}\right)}^{2}\right|+0.5{\left(\omega \;/\omega_{o}\right)}^{2}C_{1}\left(1-\cos\left[\pi \left(W/\lambda \right)\right]\right)\right\}}$$

(58) $$\gamma_{2,\Delta \sigma }=\frac{\left|1-0.02543{\left(\omega \;/\omega_{o}\right)}^{2}\right|\left\{\left|1-{\left(\omega \;/\omega_{o}\right)}^{2}\right|+\left(\frac{2}{3}\right)\left(n^{2}+3n+2\right){\left(\omega \;/\omega_{o}\right)}^{2}C_{2}\right\}}{\left|1-{\left(\omega \;/\omega_{o}\right)}^{2}\right|\left\{\left|1-0.02543{\left(\omega \;/\omega_{o}\right)}^{2}\right|+\left(\frac{1}{3}\right)\left(2^{n-3}\right)\left(n^{2}+3n+2\right){\left(\omega \;/\omega_{o}\right)}^{2}C_{2}\left(1-\cos\left[\pi \left(W/\lambda \right)\right]\right)\right\}}$$

where *C*_1_ = *P_o_ω_o_*^2^/*F* and *C*_2_ = *P_o_ω_o_*^2^(*L/d*)/[Δ*σ_o_W*(1 − *v*)].

## 3. Results and Discussion

### 3.1. Validation of the Results

**Figure 3.**
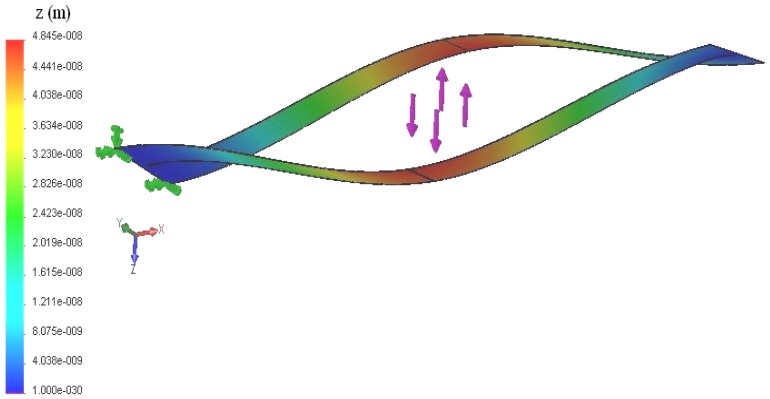
Deflection profile for rectangular microcantilever with long-slit with *L_o_* = 425 μm, *L* = 415 μm, *W* = 60 μm, *d* = 0.4 μm, *c* = 5 μm, *δ* = 2 μm, *E* = 0.1124 N μm^−2^, *v* = 0.28, and *F* = 2 × 10^−9^ N.

**Figure 4.**
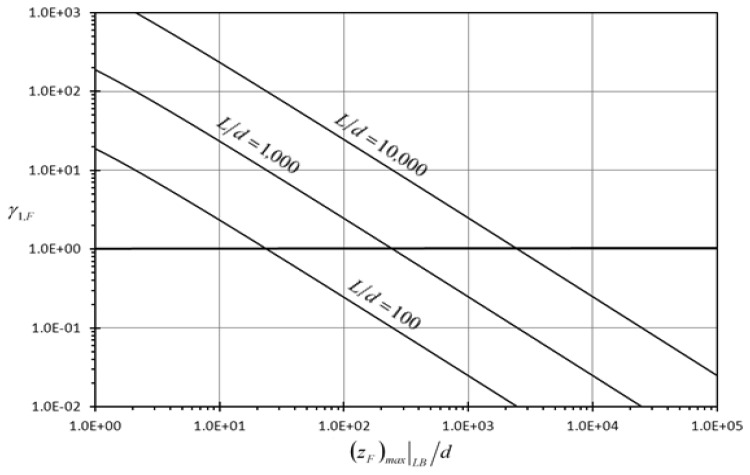
Effects of maximum rectangular microcantilever with long-slit side beams dimensionless deflection {(*z_F_*)*_max_*∣*_LB_/d*} and the slit profile dimensionless length (*L/d*) on the first detection enhancement indicator due to concentrated force loading(*γ_1,F_*).

**Figure 5.**
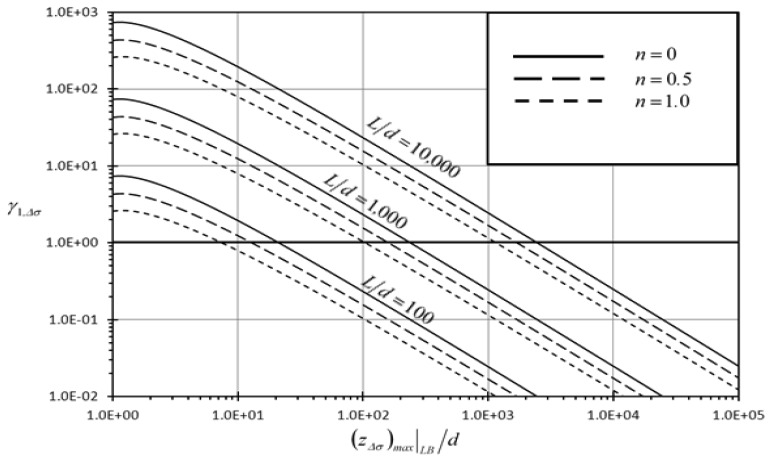
Effects of maximum rectangular microcantilever with long-slit side beams dimensionless deflection {(*z_Δσ_*)*_max_*∣*_LB_/d*}, the slit profile dimensionless length (*L/d*), and power law index (*n*) on the first detection enhancement indicator due to prescribed surface stress loading (*γ_1_*,_Δ_*_σ_*).

**Figure 6.**
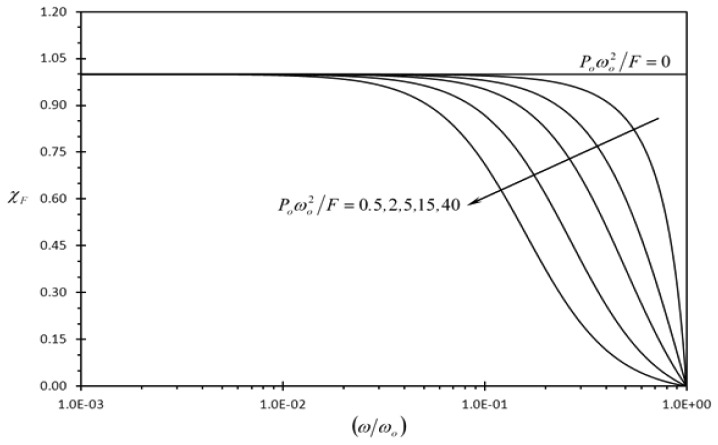
Effects of the dimensionless frequency of dynamic disturbance (*ω/ω_o_*) and the first dimensionless dynamic disturbance force amplitude {*P_o_ω_o_*^2^/*F*} on the clearness indicator of the deflection signal due to concentrated force loading (*χ_F_*).

**Figure 7.**
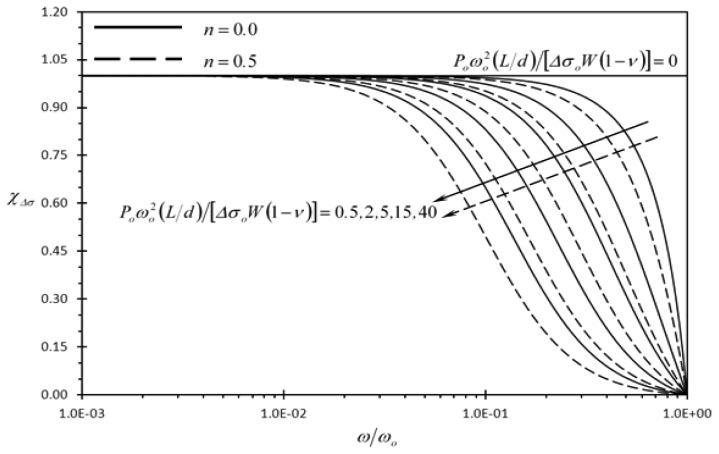
Effects of the dimensionless frequency of dynamic disturbance (*ω/ω_o_*), the second dimensionless dynamic disturbance force amplitude {*P_o_ω_o_*^2^(*L/d*)/[Δ*σ_o_W*(1 − *ν*)]}, and power law index (*n*) on the clearness indicator of the deflection signal due to prescribed surface stress loading (*χ*_Δ_*_σ_*).

**Figure 8.**
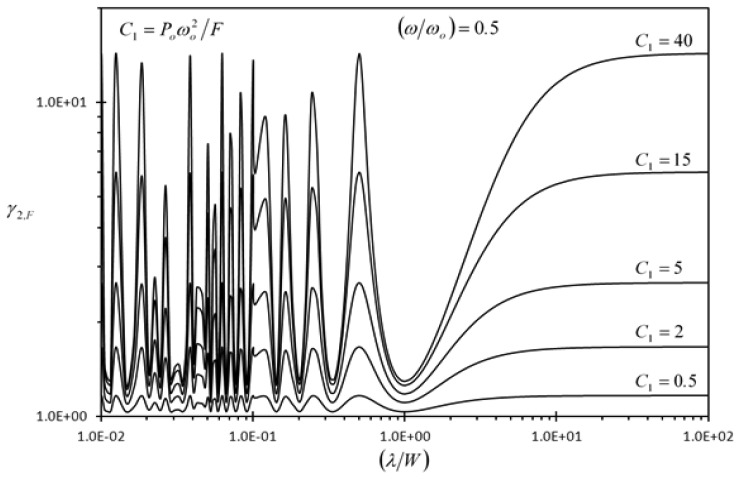
Effects of the dimensionless dynamic disturbance wavelength (*λ/W*) and the first dimensionless dynamic disturbance force amplitude {*P_o_ω_o_*^2^/*F*} on the second detection enhancement indicator of the rectangular microcantilever with long-slit due to concentrated force loading (*γ_2,F_*).

**Figure 9.**
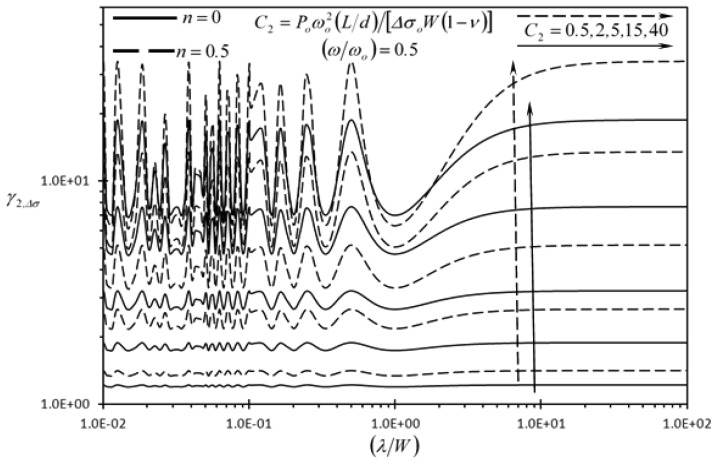
Effects of the dimensionless dynamic disturbance wavelength (*λ/W*) and the second dimensionless dynamic disturbance force amplitude {*P_o_ω_o_*^2^(*L/d*)/[Δ*σ_o_W*(1 − *ν*)]} on the second detection enhancement indicator of the rectangular microcantilever with long-slit due to prescribed surface stress loading (*γ_2_*,_Δ_*_σ_*).

**Figure 10.**
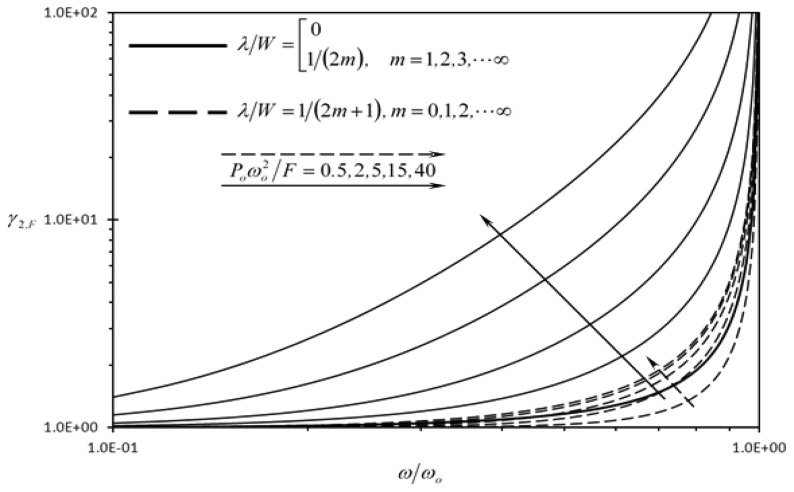
Effects of the dimensionless frequency of dynamic disturbance (*ω/ω_o_*) and the first dimensionless dynamic disturbance force amplitude {*P_o_ω_o_*^2^/*F*} on the second detection enhancement indicator of the rectangular microcantilever with long-slit due to concentrated force loading (*γ_2,F_*).

**Figure 11.**
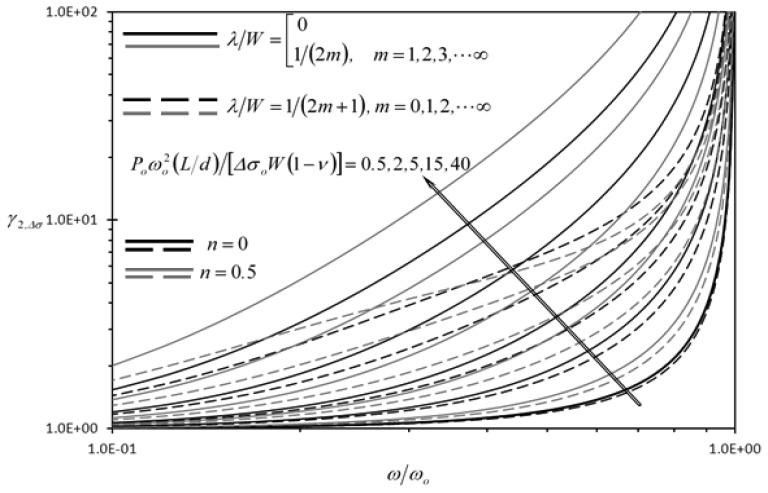
Effects of the dimensionless frequency of dynamic disturbance (*ω/ω_o_*), the power law index (*n*) and the second dimensionless dynamic disturbance force amplitude {*P_o_ω_o_*^2^(*L/d*)/[Δ*σ_o_W*(1 − *ν*)]} on the second detection enhancement indicator of the rectangular microcantilever with long-slit due to prescribed surface stress loading (*γ_2_*,_Δ_*_σ_*).

The present analytical methods for the rectangular microcantilever with long-slit were tested against an accurate numerical solution using finite element methods and accounting for all mechanical constraints induced by the geometry. Among these constraints is the torsion effect of the concentrated force and restraining the wrapping of the side beams due to the end portions of the microcantilever. The deflection contours for the present microcantilever with *L_o_* = 425 μm, *L* = 415 μm, *W* = 60 μm, *d* = 0.4 μm, *c* = 5 μm, and *δ* = 2 μm under concentrated force loading with *F* = 2 × 10^−9^ N is shown in Figure 3. The microcantilever material was taken to be silicon with *E* = 0.1124 N μm^−2^ and a poisons ratio of *v* = 0.28. The maximum deflection of LB is equal to (*z_F_*)*_max_* = 41.4 nm using Equation (31). As can be seen from Figure 2, the average deflection of the mid-section of the LB is about (*z_F_*)*_max_* = 46.4 nm. Notice that the maximum error between the numerical and the derived analytical solutions is less than 11 percent. The previous small percentage difference gives more confidence on the obtained results. The generated results of various defined detection performance indicators are presented graphically in Figures 4–11. These figures are discussed in next subsection.

### 3.2. Discussion of the Results

#### 3.2.1. Discussion of the Results of First Detection Enhancement Indicator

Figures 4 and 5 show the variation of the first detection enhancement indicator of the rectangular microcantilever with long-slit due to concentrated force loading and prescribed surface stress loading, (*γ_1,F_*, *γ_1_*,_Δ_*_σ_*) respectively, with maximum side beam relative deflection {(*z_F_*)*_max_*|*_LB_/d*} for different slit profile dimensionless length (*L/d*). It is noticed that both *γ_1,F_* and *γ_1_*,_Δ_*_σ_* increase as (*z_F_*)*_max_*|*_LB_/d* decreases and as *L/d* increases. This indicates the superiority of the rectangular microcantilever with long-slit over typical rectangular microcantilever in detection of low analyte concentration especially when very long slits are considered. As shown in Figure 4, the first detection enhancement indicator of the rectangular microcantilever with long-slit *γ_1,F_* is larger than 100 when *L/d* ≥ 1,000 and (*z_F_*)*_max_*|*_LB_/d* <2.5. The latter constraints fit almost all of microcantilever microsensor applications. As seen from Figure 5, *γ_1_*,_Δ_*_σ_* is larger than 100 when *L/d* ≥ 10,000 and (*z_Δσ_*)*_max_*|*_LB_/d* < 20. The previously mentioned constraints fit most of microcantilever applications. Therefore, both figures show that the detection capability of the rectangular microcantilever with long-slit can be more than 100 times that of the typical rectangular micocantilever. Moreover, it is noticed from Figure 4 that *γ_1_*,_Δ_*_σ_* is enhanced as *n* decreases and its maximum value occur when *n* = 0. This means that well-mixed analyte solutions produce better detection capability than weakly mixed ones as *n* index approaches *n* = 0 when the mixing level increases.

#### 3.2.2. Discussion of the Results of Clearness Indicator of Typical Rectangular Microcantilever

Figures 6 and 7 show the effect of the dimensionless frequency of dynamic disturbance (*ω/ω_o_*) on the clearness indicator of the deflection signal of the typical rectangular microcantilever due to concentrated force loading and prescribed surface stress loading (*χ_F_*, *χ*_Δ_*_σ_*), respectively. It is noticed that both *χ_F_* and *χ*_Δ_*_σ_* goes to zero as the frequency of dynamic disturbance approaches the fundamental natural frequency (*ω/ω_o_* = 1.0). This indicates that the detection of the typical microcantilever becomes unrecognizable when *ω* approaches *ω* = *ω_o_*. Moreover, both indicators are expected to decrease as the amplitude of dynamic disturbance excitation force per square of disturbance frequency (*P_o_*) increases. This behavior is noticeable in Figures 6 and 7. Furthermore, it is seen in Figure 6 that *χ*_Δ_*_σ_* is enhanced as *n* decreases and its maximum values occur when *n* = 0. Again this confirms that well-mixed analyte solutions produce lower disturbance levels in the detection signal than weakly mixed ones as *n* approaches *n* = 0 when the mixing level increases.

#### 3.2.3. Discussion of the Results of Second Detection Enhancement Indicator

Figures 8 and 9 show the effect of the dimensionless dynamic disturbance wavelength (*λ/W*) on the second detection enhancement indicator of the rectangular microcantilever with long-slit under concentrated force loading and prescribed surface stress loading (*γ_2,F_*, *γ_2_*,_Δ_*_σ_*), respectively. The results of this figure are generated with dimensionless frequency of dynamic disturbance equal to (*ω/ω_o_*) = 0.5. It is noticed that both indicators are always larger than one, regardless of the wave-length. As such, the detection of the rectangular microcantilever with long-slit is better isolated against dynamic disturbances than the typical rectangular microcantilever. When *λ/W* ≥ 3.4827, *γ_2,F_* and *γ_2_*,_Δ_*_σ_* are always increasing as the wavelength increases. For small wavelength dynamic disturbances, there is a chance that the wave disturbances at the center of LB and RB be of the same phase shift, phase shift difference of *π* or of phase shift difference between 0 and *π*. In case of the same phase shift, the disturbance in the detection of the rectangular microcantilever with long-slit is eliminated when subtracting the deflection of RB from that of LB. For the case when *π* is the phase shift difference, the subtraction process leads to agglomeration in the disturbance in the detection signal as dictated from Equations (57) and (58). When the phase shift difference is between 0 and *π*, the disturbance in the detection signal becomes more significant as the phase shift difference approaches *π*. Figures 8 and 9 demonstrate that larger dynamic disturbance forces obtained by larger *P_o_* values make the rectangular microcantilever with long-slit more superior than the typical rectangular microcantilever since *γ_2,F_* and *γ_2_*,_Δ_*_σ_* increases as *P_o_* increases. Finally, Figure 9 shows that *γ_2_*,_Δ_*_σ_* increases as *n* increases. This is expected because as *n* increases, the effective force producing the deflection decreases causing a similar effect as that of increasing the *C*_2_ value.

Figure 10 shows the effect of the dimensionless frequency of dynamic disturbance (*ω/ω_o_*) on the second detection enhancement indicator of the rectangular microcantilever with long-slit due to concentrated force loading (*γ_2,F_*) for two different sets of wavelengths. The set shown for solid lines produce same phase shift for the wave disturbances at the center of LB and RB. For this set, the clearance indicator of the rectangular microcantilever with long-slit is equal to one. That is, the detection quantities are unaffected by dynamic disturbances for this set of wavelengths. As such, *γ_2,F_* values are maxima for that set of wavelengths. On the other hand, The set of wavelengths shown for dashed lines produce phase shift difference between the wave disturbances at the center of LB and RB equal to *π*. For this set, the rectangular microcantilever with long-slit will have the minimum values of clearance indicators thus, *γ_2,F_* values are minimal for the dashed lines. According to Figure 10, *γ_2,F_* is always larger than one, regardless of the frequency of the dynamic disturbance as long as *ω* < *ω_o_*. When *ω* > *ω_o_*, the one degree of freedom model cannot be used to determine the disturbance in the deflection and more advanced models are required such as the Euler-Bernoulli beam theory [23]. The applications of these advanced models on the rectangular microcantilever with long-slit have many complications due to the complexity of the geometry. Moreover, *γ_2,F_* is always increasing as *ω* increases. Similar trends are shown in Figure 11, where the rectangular microcantilever with long-slit is under the prescribed surface stress loading. Furthermore, the superiority of the rectangular microcantilever with long-slit over the typical rectangular microcantilever increases as *P_o_* increases is shown in Figures 10 and 11, since both *γ_2,F_* and *γ_2_*,_Δ_*_σ_* increase as *P_o_* increases.

## 4. Conclusions

An investigation verifying the advantage of using rectangular microcantilevers with long-slits in microsensing applications was performed in this work, based on analytical solutions. The detection capabilities of these microcantilevers were compared against that of typical rectangular microcantilevers under the action of dynamic disturbances. Concentrated force loadings and prescribed surface stress loadings were considered as the sensing driving forces. The theory of linear elasticity for thin beam deflections was used to obtain the detection quantities. The disturbance in these quantities was obtained using the wave propagation and beam vibration theories. The defection profile of the rectangular microcantilever with long-slit was validated against an accurate numerical solution utilizing finite element method with a maximum deviation less than 11 percent.

It was found that the detection of rectangular microcantilevers with long-slits based on their maximum slit opening length can be more than 100 times the maximum deflection of the typical rectangular microcantilever. Furthermore, the disturbance (noise) in the deflection of the microcantilever with long-slit was found to be always smaller than that of the typical microcantilevers, regardless of the wavelength, force amplitude, and the frequency of the dynamic disturbance. Moreover, good mixing the analyte solution was found to produce better detection capability and smaller disturbance in the detection of the microcantilever with long-slit than weakly-mixed ones. Eventually, detections of the microcantilevers with long-slit were found to be practically unaffected by dynamic disturbances as long as the wavelengths of these disturbances are larger than 3.5 times the width of the microcantilever. Finally, the present work strongly suggests implementation of microcantilevers with long-slit as microsensors in real analyte environments and out of the laboratory testing.

## Figures and Tables

**Table 1.** Maximum value of rectangular microcantilever with long-slit side beams deflection that produces detection enhancement indicator due to concentrated force loading larger than unity.

(*d/L*)	10^−2^	10^−3^	10^−4^	10^−5^
[(*z_F_*)*_max_*∣*_LB_/d*]*_γF_*_=1_	23.928	246.81	2,490.1	24,969
[(*z_F_*)*_max_*∣*_LB_/d*]*_γF_*_=1_ = 0.23467 ×exp(−1.00593×*d/L*)				

**Table 2.** Maximum value of rectangular microcantilever with long-slit side beams deflection that produces detection enhancement indicator due to prescribed differential surface stress loading larger than unity.

*n*	(*d/L*)	[(*z_Δσ_*)*_max_*∣*_LB_/d*]∣*_γΔσ_*_=1_	*n*	(*d/L*)	[(*z_Δσ_*)*_max_*|*_LB_/d*]∣*_γΔσ_*_=1_
*n* = 0	10^−2^	21.157	*n* = 1.0	10^−2^	7.4114
	10^−3^	238.55		10^−3^	103.90
	10^−4^	2,464.4		10^−4^	1155.5
	10^−5^	24,888		10^−5^	12067
*n* = 0.5	10^−2^	12.854	*n* = 1.5	10^−2^	3.9373
	10^−3^	159.14		10^−3^	66.535
	10^−4^	1,699.3		10^−4^	775.55
	10^−5^	17,408		10^−5^	8288.2

## Nomenclature

*c*: clearance length for rectangular microcantilever with long-slit (μm)
*d*: microcantilever thickness (μm)
*E*: elastic modulus (N μm^−2^)
*F*: concentrated force (N)
*I*: area moment of inertia (μm^4^)
*k*: stiffness (N/μm)
*L*: typical rectangular microcantilever or slit length (μm)
*L_o_*: length of rectangular microcatilever with long-slit (μm)
*M*: moment (N μm)
*m*: mass (kg)
*n*: surface stress model index
*P_o_*: dynamic disturbance force per square of frequency of disturbance (N s^−2^)
*t*: time variable (s)
*W*: total microcantilever width (μm)
*x*: axis of the extension dimension (μm)
*Y*: effective elastic modulus (N μm^−2^)
*Z*: first deflection indicator
*z*: deflection (μm)
*z_d_*: Amplitude of disturbance in deflection (μm)

**Greek Symbols**
*χ*: detection clearness indicator
*δ*: slit thickness (μm)
*γ_1_*: the first detection enhancement indicator
*γ_2_*: the second detection enhancement indicator
*λ*: wavelength of the dynamic disturbance (μm)
*ν*: Poisson's ratio
*σ*: surface stress (N μm^−1^)
*ω*: dynamic disturbance frequency (s^−1^)
*ω_o_*: first natural frequency of typical rectangular microcantilever (s^−1^)
*ω_s_*: first natural frequency of rectangular microcantilever with long-slit (s^−1^)

**Subscripts**
*d*: disturbance
*F*: concentrated force condition
Δ*σ*: prescribed surface stress condition
*eff*: effective value

**Abbreviations**
*LB*: left beam of the rectangular microcantilever with long-slit
*RB*: right beam of the rectangular microcantilever with long-slit

## References

[1] Alkamine S., Barrett R.C., Quate C.F. (1990). Improved atomic force microscope images using microcantilevers with sharp tips. Appl. Phys. Lett..

[2] Arntz Y., Seelig J.D., Lang H.P., Zhang J., Hunziker P., Ramseyer J.P., Meyer E., Hegner M., Gerber C. (2003). Label-free protein assay based on a nanomechanical cantilever array. Nanotechnology.

[3] Suri C.R., Kaur J., Gandhi S., Shekhawat G.S. (2008). Label-free ultra-sensitive detection of atrazine based on nanomechanics. Nanotechnology.

[4] Calleja M., Nordstrom M., Alvarez M., Tamayo J., Lechuga L.M., Boisen A. (2005). Highly sensitive polymer-based cantilever-sensors for DNA detection. Ultramicroscopy.

[5] Wu G., Ji H., Hansen K., Thundat T., Datar R., Cote R., Hagan M.F., Chakraborty A.K., Majumdar A. (2001). Origin of nanomechanical cantilever motion generated from biomolecular interactions. Proc. Natl. Acad. Sci. USA.

[6] Khaled A.-R.A., Vafai K., Yang M., Zhang X., Ozkan C.S. (2003). Analysis, control and augmentation of microcantilever deflections in bio-sensing systems. Sens. Actuators B.

[7] Boisen A., Thaysen J., Jensenius H., Hansen O. (2000). Environmental sensors based on micromachined cantilevers with integrated read-out. Ultramicroscopy.

[8] Zuo G., Li X., Zhang Z., Yang T., Wang Y., Cheng Z., Feng S. (2007). Dual-SAM functionalization on integrated cantilevers for specific trace-explosive sensing and non-specific adsorption suppression. Nanotechnology.

[9] Zhang G., Zhao L., Jiang Z., Yang S., Zhao Y., Huang E., Hebibul R., Wang X., Liu Z. (2011). Suface stress-induced deflection of a microcantilver with various widths and overall microcantilever sensitivity enhancement via geometry modification. J. Phys. D Appl. Phys..

[10] Fritz J., Baller M.K., Lang H.P., Rothuizen H., Vettiger P., Meyer E., Güntherodt H.-J., Gerber C., Gimzewski J.K. (2000). Translating biomolecular recognition into nanomechanics. Science.

[11] Lee H.J., Chang Y.S., Lee Y.P., Jeong K.-H., Kim H.-Y. (2007). Deflection of microcantilever by growing vapor bubble. Sens. Actuators A.

[12] Jeon S., Jung N., Thundat T. (2007). Nanomechanics of self-assembled monolayer on microcantilever sensors measured by a multiple-point deflection technique. Sens. Actuators B.

[13] Yang M., Zhang X., Vafai K., Ozkan C.S. (2003). High sensitivity piezoresistive cantilever design and optimization for an analyte-receptor binding. J. Micromech. Microeng..

[14] Vafai K., Ozkan C., Haddon R., Khaled A.-R.A., Yang M. (2007). Microcantilevers for Biological and Chemical Assays and Methods of Making and Using Thereof.

[15] Zhu Q., Shih W.Y., Shih W.-Y. (2008). Enhanced detection resonance frequency shift of a piezoelectric microcantilever sensor by a DC bias electric field in humidity detection. Sens. Actuators B.

[16] Yen Y.-K., Huang C.-Y., Chen C.-H., Hung C.-M., Wu K.-C., Lee C.-K., Chang J.-S., Lin S., Huang L.-S. (2009). A novel, eclectically protein-manipulated microcantilever biosensor for enhancement of capture antibody immobilization. Sens. Actuators B.

[17] Ansari M.Z., Cho C. (2009). Deflection, Frequency, and Stress Characteristics of Rectangular, Triangular, and Step Profile Microcantilevers for Biosensors. Sensors.

[18] Vafai K., Khaled A.-R.A. (2010). Innovative biosensors for chemical and biological assays.

[19] Khaled A.-R.A., Vafai K. (2004). Optimization modelling of analyte adhesion over an inclined microcantilever-based biosensor. J. Micromech. Microeng..

[20] Khanafer K., Khaled A.-R.A., Vafai K. (2004). Spatial optimization of an array of aligned microcantilever based sensors. J. Micromech. Microeng..

[21] Khanafer K., Vafai K. (2005). Geometrical and flow configurations for enhanced microcantilever detection within a fluidic cell. Int. J. Heat Mass Transf..

[22] Vafai K., Khaled A.-R.A. (2010). Methods and devices comprising flexible seals, flexible microchannels, or both for modulating or controlling flow and heat.

[23] Khaled A.-R.A., Vafai K. (2011). Analysis of Deflection Enhancement Using Epsilon Assembly Microcantilevers Based Sensors. Sensors.

[24] Rao S.S. (2010). Mechanical Vibrations.

[25] Sader J. (1998). Frequency response of cantilever beams immersed in viscous fluids with applications to the atomic force microscope. J. Appl. Phys..

